# Correction: Theory to Predict Shear Stress on Cells in Turbulent Blood Flow

**DOI:** 10.1371/journal.pone.0117894

**Published:** 2015-02-03

**Authors:** The PLOS ONE Staff

## Notice of Republication

This article was republished on January 1, 2015, to correct errors in equations that were introduced during the typesetting process. Please download this article again to view the correct version. The originally published, uncorrected article and the republished, corrected article are provided here for reference.

## Supporting Information

S1 File Originally published, uncorrected article. (PDF)

S2 File Republished corrected article. (PDF)
