# Supplementary material for: Correction: Theory to Predict Shear Stress on Cells in Turbulent Blood Flow
Source: PLoS One. 2015 Feb 3;10(2):e0117894. doi: 10.1371/journal.pone.0117894 (PMC4315595; doi:10.1371/journal.pone.0117894)
Supplement: S2 File — (PDF) [file pone.0117894.s002.pdf]

OPEN ACCESS Freely available online

PLOS ONE

Theory to Predict Shear Stress on Cells in Turbulent Blood Flow

Khandakar Niaz Morshed[1], David Bark Jr.[1,2], Marcio Forleo[2], Lakshmi Prasad Dasi[1,2]*

1 Department of Mechanical Engineering, Colorado State University, Fort Collins, Colorado, United States of America, **2** School of Biomedical Engineering, Colorado State University, Fort Collins, Colorado, United States of America

Abstract

Shear stress on blood cells and platelets transported in a turbulent flow dictates the fate and biological activity of these cells. We present a theoretical link between energy dissipation in turbulent flows to the shear stress that cells experience and show that for the case of physiological turbulent blood flow: (a) the Newtonian assumption is valid, (b) turbulent eddies are universal for the most complex of blood flow problems, and (c) shear stress distribution on turbulent blood flows is possibly universal. Further we resolve a long standing inconsistency in hemolysis between laminar and turbulent flow using the theoretical framework. This work demonstrates that energy dissipation as opposed to bulk shear stress in laminar or turbulent blood flow dictates local mechanical environment of blood cells and platelets universally.

Citation: Morshed KN, Bark D Jr, Forleo M, Dasi LP (2014) Theory to Predict Shear Stress on Cells in Turbulent Blood Flow. PLoS ONE 9(8): e105357. doi:10.1371/journal.pone.0105357

Editor: Dermot Cox, Royal College of Surgeons, Ireland

Received January 8, 2014; **Accepted** July 23, 2014; **Published** August 29, 2014

Copyright: © 2014 Morshed et al. This is an open-access article distributed under the terms of the Creative Commons Attribution License, which permits unrestricted use, distribution, and reproduction in any medium, provided the original author and source are credited.

Funding: Funding from National Institutes of Health (NIH) under Award Number R01HL119824, and the American Heart Association under award 11SDG5170011. The funders had no role in study design, data collection and analysis, decision to publish, or preparation of the manuscript.

Competing Interests: The authors have declared that no competing interests exist.

* Email: lakshmi.dasi@colostate.edu

Introduction

Turbulence is ubiquitous, and is particularly prominent in engineered cardiovascular devices as well as pathophysiological blood flow. There is strong evidence that turbulence impacts the environments of erythrocytes and platelets on the cellular level [1,2] in a manner physically distinct from that known in simple laminar shear flows such as in a viscometer. While our physical understanding of the structure of turbulence and its universal properties has received significant attention in the past [3–7], there is no theory that links the statistical properties of turbulence to shear stresses physically experienced by cells transported in whole blood flow. Shear stress acting on cell membranes is a critical mechanical cue that regulates biological activity [7–10] and therefore a theory relating turbulence to shear stress environment of cells is necessary.

In turbulent blood flow, the complex spatio-temporal fluctuations of shear stress leads to hemolysis and platelet activation [11,12]. Such phenomena are critical when designing life saving devices such as artificial hearts, ventricular assist devices, stents, grafts, and heart valves. The phenomena can further impact disease such as in the case of an aortic stenosis or atherosclerosis. Current models that predict stress experienced by blood cells are purely empirical and based on classic experiments [12] that yield paradoxical results under laminar and turbulent conditions [13]. For a comprehensive review of studies on turbulent blood flow related hemolysis and platelet activation, the reader is directed to Refs [12,14–17]. As discussed in Ref [12], despite many of these studies, there has not been a solid physically justifiable connection made between turbulence and the shear stress acting on blood cells and platelets. A strong requirement of a physical theory is that it should make a link, in a manner independent of laminar and turbulent regimes of flow because the pertinent parameter is flow at the length scale of individual cells, where it is considered laminar. Another aspect that is important to consider is the notion of universality of turbulent structures despite the intermittency issue [6]. Do the universality properties of turbulence hold in the most complex of blood flows considering Newtonian and non-Newtonian properties of blood? If so, will the distribution of shear stress acting on blood cells and platelets be universal? Here the term "universal" is used not to imply homogeneous isotropic turbulence (HIT), but rather loosely to emphasize the significant agreement between the distributions of instantaneous dissipative scales in complex in-homogeneous shear flows with the distributions observed in HIT as well as the robust presence of inertial range scaling[18–21].

The goal of this work is to address the above by introducing a unified (in the sense of laminar vs. turbulent) physical theory to: (1) identify the relevant dynamic properties of flow that link to the predicted shear stress experience by cells based on fundamental physical arguments. We achieve this for the special case of blood, but the underlying physical arguments may hold for any cell suspension constrained to the Newtonian regime of its rheology, (2) theoretically consider the relevance of non-Newtonian effects on the smallest scale turbulent structures for blood based on order of magnitude arguments; and (3) test the "universality" of small scale structure of turbulent flows in one of the most complex turbulent blood flow problems (flow through a bileaflet mechanical heart valve) and experimentally examine the universality of the predicted shear stress distribution, thus testing the robustness of the theory. Since we lack the capability to experimentally measure shear stress on individual cells transported in a turbulent flow, we use the new theoretical framework to resolve inconsistency in published hemolysis data in laminar and turbulent pipe flow as a

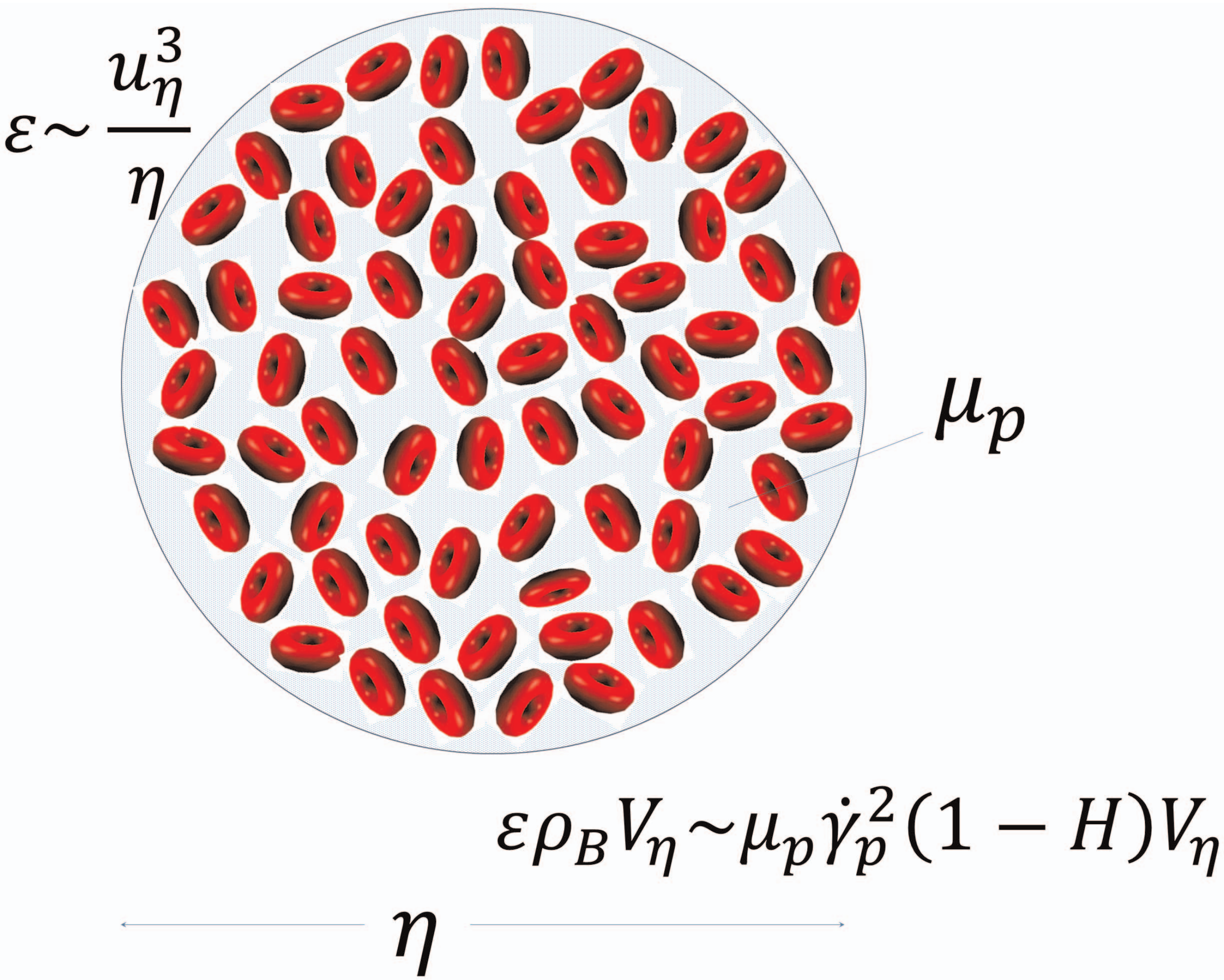

Figure 1. Schematic of blood cells in a turbulent "eddy".
doi:10.1371/journal.pone.0105357.g001

surrogate validation of the theory. Finally, we note that the theoretical framework presented in this work is not limited only to blood cells, but applies to any case of suspended cells that are sufficiently smaller than the smallest scales of turbulent motion.

Methods: Theoretical Construction

Theoretical development is initially constrained to turbulent flows where the smallest turbulent eddies are greater than the size of the red blood cell (RBC), i.e. the instantaneous minimum size of turbulent eddy is always >O(10 μm). This is an important limit, as it is not possible to physically represent turbulent eddies smaller than the size of the biggest cells with the single-phase approximation of blood. In fact, the single-phase approximation may very well be susceptible to errors even when local eddies are within two or three times the size of the red-blood cell, i.e. ~25 μm. For now, let us accept this as a limitation and later discuss repercussions of this assumption. Nevertheless, for turbulent eddies >25 μm, the single-phase continuum representation of blood may be considered valid [12]. It is also important to underscore that using a continuum single-phase model of blood cannot be equated to the assumption of RBCs passively seeded on to a continuous medium. On the contrary, the single-phase representation of blood "lumps" all effects of the physical reality into the constitutive rheology of blood.

The theoretical construction is next focused on whether small scale (dissipative) turbulent structures may be considered Newtonian or non-Newtonian. This is done through order of magnitude arguments combined with existing knowledge about blood rheology. Subsequently, we introduce basic arguments with respect to the nature of turbulent scales of motion in turbulent blood flows expected in the cardiovascular system and artificial devices. Finally, physical arguments are introduced that link turbulence statistics to the distribution of laminar shear stresses acting directly on cells.

Newtonian vs Non-Newtonian

It is well known that whole blood significantly deviates from Newtonian behavior when local bulk shear rates are below the order of 100 s^{-1} [22–24] or when a vessel diameter is less than 100 μm [25]. Now, consider the instantaneous turbulent dissipative length scale, η, defined such that the local instantaneous velocity increment (difference), δu_η, across two points in space separated by η is such that the local Reynolds number defined as

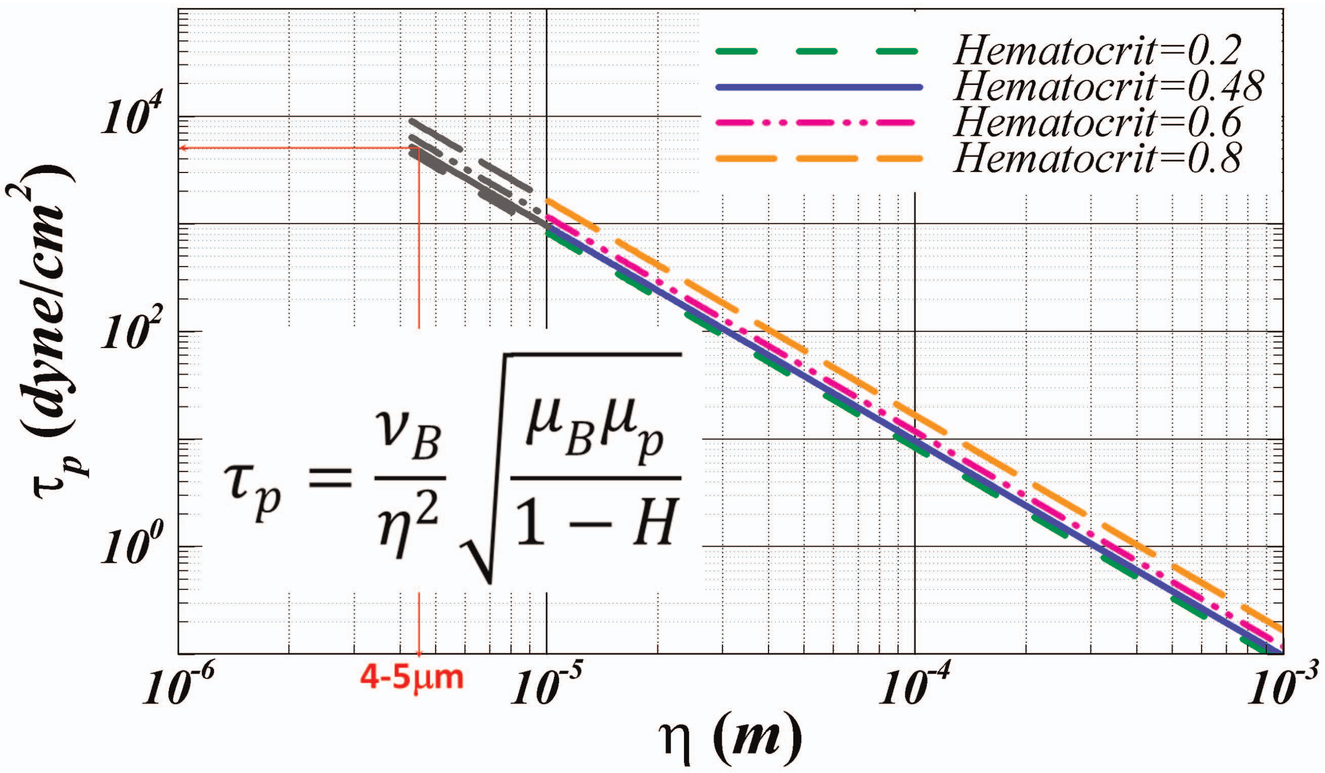

Figure 2. Shear Stress (τ_p) as a function of eddy size η plotted for increasing hematocrit based on the equation derived using energy balance.
doi:10.1371/journal.pone.0105357.g002

$\frac{\eta \delta u_\eta}{\nu} \sim 1$. This instantaneous length scale is the definition of the local turbulent dissipative scale and may be regarded as an "instantaneous" Kolmogorov scale [18–20,24,26,27]. Note that no assumptions of local isotropy or homogeneous turbulence are being made in this framework. For such a small dissipative length scale, which in a way is the true measure of the actual size of the smallest eddy, the shear rate within such an eddy can be estimated as $\dot{\gamma}_\eta \sim \frac{\delta u_\eta}{\eta} \sim \frac{\nu}{\eta^2}$. Thus the shear rate within such an eddy is set purely by ν and η. Now, non-Newtonian behavior of blood dictates that ν is dependent on $\dot{\gamma}_\eta$, which is physiologically O(1) cSt. It is easy to show that the range of $\dot{\gamma}_\eta \sim \frac{\nu}{\eta^2}$ corresponding to eddies of sizes $10\mu m < \eta < 100\mu m$ is $O(10^2 s^{-1}) < \bar{\gamma}_\eta < O(10^5 s^{-1})$. Based on these order of magnitude estimates, it follows that instantaneous turbulent eddies in the range $10\mu\mathrm{m} < \eta < 100\mu\mathrm{m}$ in any cardiovascular blood flow will always be in the Newtonian regime considering $\dot{\gamma}_\eta > O(100 s^{-1})$. In other words, any consequence or damage occurring to the cells within these eddies happens while the eddy itself behaves as a Newtonian fluid.

Scales of Motion

With the notion of blood being Newtonian for turbulent eddies 10 μm and above, let us examine the scales of turbulent motion for the smallest scales, without the assumption of isotropy or homogeneity. Recall that turbulent flow is characterized by complex spatio-temporal fluctuations, u_i, superimposed with the mean velocity field, U_i. These spatio-temporal fluctuations have been phenomenologically represented as turbulent eddies of varying length scales. It is important to clarify that physically there are no circular "eddies", but the term eddies only refer to the Fourier analogy of representing the fluctuating field as an summation of kinetic energy over "eddies" ranging from the smallest possible scales to the integral length scale. The turbulent kinetic energy (per unit mass), $k = \frac{1}{2}\langle u_i u_i \rangle$, is transferred across from large to the small scales (so called energy cascade) with viscous dissipation occurring at the smallest eddies. The Kolmogorov length scale, $\eta_K \equiv \left(\nu^3 / \langle \epsilon \rangle\right)^{1/4}$, where ν is the kinematic viscosity and ε is the average local kinetic energy dissipation rate (per unit mass), corresponds to the characteristic eddy size based on ε. The velocity scale of this eddy equals $u_{\eta_K} = \nu/\eta_K$, and therefore the Reynolds number, $\frac{u_{\eta_K}\eta_K}{\nu}$ of the Kolmogorov eddy is unity. In the context of blood damage, several studies have focused on the relevance of the Kolmogorov eddy to predict the capacity of turbulent flow to damage blood cells (reviewed in [12]). While η_K in theory represents the *average* size of the dissipative eddies (for the case of HIT), it does not, in itself, reflect the smallest eddy in a turbulent flow in realistic turbulent flows. In fact, there exist eddies that are a fraction of the Kolmogorov eddy, so called sub-Kolmogorov eddies, arising from the inherently intermittent nature of the instantaneous energy dissipation rate field [5,6]. Intermittency is a property of all turbulent flows which broadly refers to the fluctuations of the fluctuating velocity gradient tensor $s_{ij} \equiv \frac{1}{2}\left(\partial u_i/\partial x_j + \partial u_j/\partial x_i\right)$ and consequently for $\varepsilon = 2\nu s_{ij} s_{ij}$. With energy dissipation rates proportional to the square of velocity gradient tensor, the fluctuations in ε

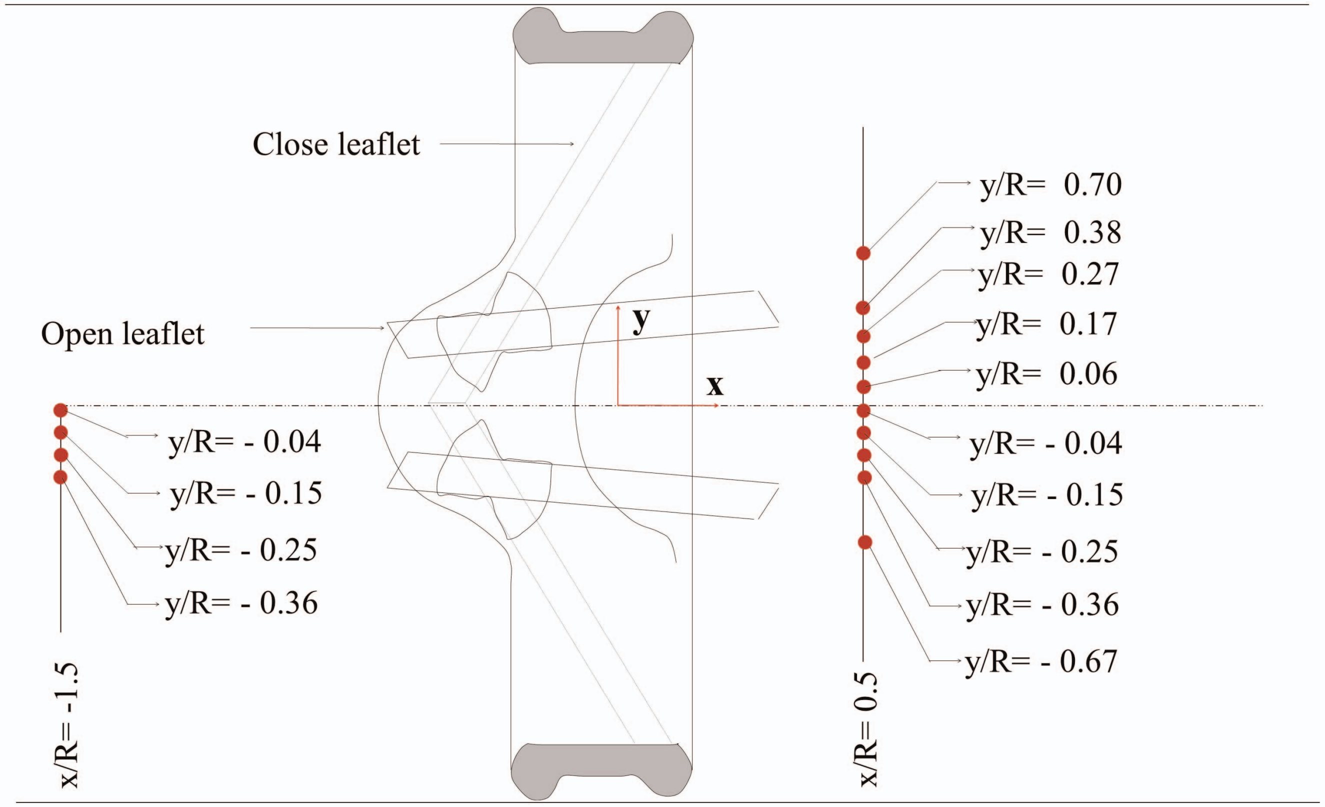

Figure 3. Schematic of the valve and points of interest upstream and downstream of the valve.
doi:10.1371/journal.pone.0105357.g003

are far greater and intense, with very large departures from its average. Physically these large positive fluctuations in energy dissipation occur due to local vortex stretching that generate momentarily intense shear regions over very small length scales, much smaller than the Kolmogorov scale itself. In other words, when the energy dissipation rate is locally above the mean energy dissipation magnitude, the corresponding eddy size is sub-Kolmogorov. Thus, the true depiction of micro-environment of cells in realistic turbulent flows corresponds to their tumultuous experiences in a spectrum of dissipative eddies ranging from sub-Kolmogorov scales all the way to the Taylor micro-scale that demarks the largest of the dissipative eddies [6]. Recent turbulence literature estimate the smallest sub-Kolmogorov scales to be of roughly $Re_L^{1/4}$ times smaller than the Kolmogorov scale [23,26,28]. Here $Re_L = \frac{k^{1/2}L}{\nu}$ is the local turbulence Reynolds number defined by the integral length scale, L, and the local turbulent kinetic energy. To date, studies addressing turbulence in blood flow have not taken into consideration the issue of intermittency or the sub-Kolmogorov fluctuations thereby ignoring the most damaging aspects of turbulent flow. In this study, we will consider sub-Kolmogorov fluctuations using direct measurements through the definition of the instantaneous turbulent dissipative length scale, η[18–20,24,26,27], while constraining our analysis only to flows where the smallest sub-Kolmogorov $\eta > 10\mu m$.

Local Energy Dissipation and Shear Stress Acting on Cells. Based on the above picture of scale distributions without invoking assumptions of isotropy or homogeneity, and introducing the notion of sub-Kolmogorov scales, it is now possible to consider a link between macroscopic properties of blood dynamics or eddy scales to the local shear stress acting on blood cells. Figure 1 illustrates a schematic of RBCs in a hypothetical dissipative scale eddy of size η. Recall that the velocity scale of this eddy is $\delta u_\eta = \frac{\nu}{\eta}$ to yield the condition of locally unit Reynolds number. Given the length scale and velocity scale, the instantaneous energy dissipation rate corresponding to this smallest eddy is $\frac{\delta u_\eta^3}{\eta} = \frac{\nu^3}{\eta^4}$.

Looking at Figure 1, let us now assume that almost all of this energy dissipation physically occurs through viscous straining of plasma fluid between cells and that the rate of energy lost in lysing or activating cells is negligible in comparison to heat generation within plasma. This is a valid assumption if (a) it is known that a very small fraction of cells lyse/activate per unit time, and (b) the strain rate of cell membrane deformation is much smaller (by at least an order of magnitude) than the strain rate of plasma surrounding the cell. The relative difference in the strain rates of the cell membrane and the surrounding plasma represents the efficiency of energy transfer from the fluid to strain-energy stored into the cell membrane. Both (a) and (b) seem valid as hemolysis/activation in most device and physiological flows occur over prolonged duration and the magnitude of free hemoglobin released is relatively very small compared to the total hemoglobin. In particular, the second condition is valid for cases when blood cells are lysed over a prolonged exposure to shearing forces as opposed to instantaneous lysis which has been observed for whole blood shear stresses >450 Pa [29,30]. Complex cardiovascular devices such as the bileaflet mechanical heart valves imposes shear

Table 1. Basic Turbulence Parameters Upstream.

	y/R	k (m^2/s^2)	L (μm)	Re_L	η_0 (μm)	η_K (μm)
**Acceleration**	−0.04	8.06E-03	521	13.35	81	75
	−0.15	8.81E-03	561	15.05	80	73
	−0.25	7.83E-03	580	14.66	84	77
	−0.36	9.24E-03	485	13.33	75	70
**Peak**	−0.04	5.25E-03	416	8.61	88	83
	−0.15	4.76E-03	283	5.58	82	78
	−0.25	5.39E-03	338	7.09	82	78
	−0.36	5.50E-03	303	6.42	79	75
**Deceleration**	−0.04	8.38E-03	512	13.38	79	73
	−0.15	9.04E-03	447	12.14	74	69
	−0.25	7.40E-03	491	12.07	81	76
	−0.36	7.43E-03	595	14.64	86	79
**Regurgitation**	−0.04	7.53E-03	365	9.06	74	70
	−0.15	9.11E-03	285	7.77	65	61
	−0.25	7.48E-03	346	8.56	74	69
	−0.36	7.57E-03	317	7.89	72	67
**Diastole**	−0.04	9.46E-02	7396	650.03	70	57
	−0.15	9.55E-02	4004	353.50	59	49
	−0.25	9.60E-02	7438	658.48	70	57
	−0.36	5.60E-02	2746	185.69	64	55

doi:10.1371/journal.pone.0105357.t001

stresses in the range <15 Pa [14]. Furthermore, (b) is supported by the relatively low energy dissipation for both cytoplasm and membrane deformation in a RBC tank treading in flow, corresponding to $O(10^{-15}$–10^{-13} watts) for shear rates $O(10^2)$, which extrapolates to $O(10^{-8}$ watt) for shear rates reaching $10^5\ s^{-1}$ [31,32].

Given these assumptions, which appear reasonably valid, the rate of energy dissipated within the eddy shown in Figure 1, ε, may be approximated to the rate of energy dissipated in the fluid that surrounds cells. The following equation represents the energy balance in watts:

$$\varepsilon \rho_B V_\eta \sim \mu_p \dot{\gamma}_p^2 (1-H) V_\eta \quad (1)$$

Where ρ_B is the density of whole blood, V_η is the volume of the eddy, μ_p is the dynamic viscosity of plasma, $\dot{\gamma}_p$ is the strain rate in the plasma surrounding blood cells, and $0<H<1$ is the fractional hematocrit. It is easy to verify that both sides of the equation have units of power. In the above equation, if the plasma dynamic viscosity is known, then it is straight forward to estimate the viscous shear stress, $\tau_p = \mu_p \dot{\gamma}_p$, within the plasma surrounding the cells as a function of energy dissipation rate. Strictly, the cells experience τ_p, not τ_B which can be estimated as $\tau_B = \mu_B \dot{\gamma}_B = \mu_B \frac{v_B}{\eta^2}$. Clearly, it is evident from a physical basis that the relevant dynamical property of blood flow that dictates τ_p is the local energy dissipation rate, ε.

Recognizing that it is easier to measure ηrather than ε, we solve equation 1 for $\tau_p = \mu_p \dot{\gamma}_p$ after substituting $\varepsilon = \frac{v_B^3}{\eta^4}$ to give:

$$\tau_p = \frac{v_B}{\eta^2} \sqrt{\frac{\mu_B \mu_p}{(1-H)}} \quad (2)$$

The above equation relates the instantaneous dissipative scale η in a general turbulent blood flow to the corresponding shear stress acting on cells. It is important to note that τ_p and τ_B are off by a factor $\tau_p/\tau_B = \left[\frac{\mu_p}{\mu_B(1-H)}\right]^{1/2}$. For typical values, τ_p is about 80% of τ_B but may easily exceed τ_B if locally$H > 1 - \frac{\mu_p}{\mu_B}$. Local variations in hematocrit under turbulent straining may produce large discrepancies between τ_p and τ_B.

Figure 2 presents the theoretical estimate of shear stress τ_p using equation 2 over a range of turbulent eddy sizes $10\mu m < \eta < 10^3 \mu m$ and hematocrit $0<H<0.8$. As shown in this figure, τ_p ranges 10^{-1}–10^5 $dyne/cm^2$. We must note here that we extrapolate equation 1 for η as low as half the size of the RBC. It is interesting to note that for an η about 5–6 μm, equation 1 predicts a shear stress between 400–500 N/m^2, a magnitude well known to instantly lyse RBCs [29,30]. This illustrates that the τ_p prediction of equation 1 up η approaching 10 μm appears to asymptotically reach the instantaneous lysis value of 400–500 N/m^2 [29,30]. Furthermore these shear stresses correspond to the release of serotonin from platelets, demonstrating granule release, a process involved in platelet activation [33].

In real turbulent flows, η is a dynamically fluctuating quantity in space and time, around the statistical measure $\eta_K \equiv \left(v_B^3/\langle\epsilon\rangle\right)^{1/4}$. Substituting η_K in equation 2 will only represent an average shear

Table 2. Basic Turbulence Parameters Downstream.

	y/R	k (m^2/s^2)	L (μm)	Re_L	η_0 (μm)	η_k (μm)
**Acceleration**	0.70	5.07E-03	669	13.61	102	94
	0.38	2.70E-02	1558	73.08	71	62
	0.27	2.16E-02	1543	64.81	77	68
	0.17	1.54E-02	1875	66.48	91	81
	0.06	9.70E-03	903	25.42	88	80
	−0.04	1.23E-02	1474	46.70	93	83
	−0.15	2.09E-02	1651	68.12	79	70
	−0.25	2.11E-02	2252	93.53	86	75
	−0.36	2.16E-02	1183	49.68	71	63
	−0.67	1.60E-02	484	17.49	62	57
**Peak**	0.70	3.32E-03	255	4.21	91	87
	0.38	1.09E-01	3537	333.25	54	45
	0.27	9.38E-02	2740	239.76	53	45
	0.17	9.84E-02	2907	260.54	53	45
	0.06	5.75E-02	2212	151.56	60	51
	−0.04	5.67E-02	1833	124.73	57	49
	−0.15	1.16E-01	3873	376.51	54	45
	−0.25	9.54E-02	2321	204.83	50	43
	−0.36	1.05E-01	2159	199.75	47	41
	−0.67	1.13E-02	423	12.88	67	62
**Deceleration**	0.70	4.70E-03	719	14.10	107	99
	0.38	2.61E-02	2009	92.67	77	67
	0.27	2.30E-02	2163	93.81	82	72
	0.17	2.54E-02	1273	57.99	68	61
	0.06	2.53E-02	2004	91.04	78	68
	−0.04	2.76E-02	2021	95.91	76	66
	−0.15	2.33E-02	1468	63.99	74	65
	−0.25	2.41E-02	1369	60.69	71	63
	−0.36	2.21E-02	1701	72.27	78	69
	−0.67	1.70E-02	1122	41.76	76	68
**Regurgitation**	0.70	8.42E-03	269	7.05	66	62
	0.38	1.00E-02	10392	296.89	172	145
	0.27	9.61E-03	9143	256.12	168	143
	0.17	9.17E-03	12616	345.11	188	158
	0.06	8.35E-03	7423	193.75	167	143
	−0.04	9.18E-03	4613	126.30	142	122
	−0.15	7.72E-03	16109	404.49	213	179
	−0.25	7.63E-03	8359	208.61	179	152
	−0.36	6.40E-03	6622	151.36	178	153
	−0.67	2.23E-03	888	11.98	148	138
**Diastole**	0.70	3.53E-03	376	6.39	99	94
	0.38	1.00E-02	10392	8.16	94	89
	0.27	9.61E-03	9143	7.40	93	88
	0.17	9.17E-03	12616	6.42	96	91
	0.06	8.35E-03	7423	5.33	98	93
	−0.04	9.18E-03	4613	4.68	95	90
	−0.15	7.72E-03	16109	5.66	103	98
	−0.25	7.63E-03	8359	5.96	105	100
	−0.36	6.40E-03	6622	4.24	103	99
	−0.67	2.23E-03	888	2.15	110	108

doi:10.1371/journal.pone.0105357.t002

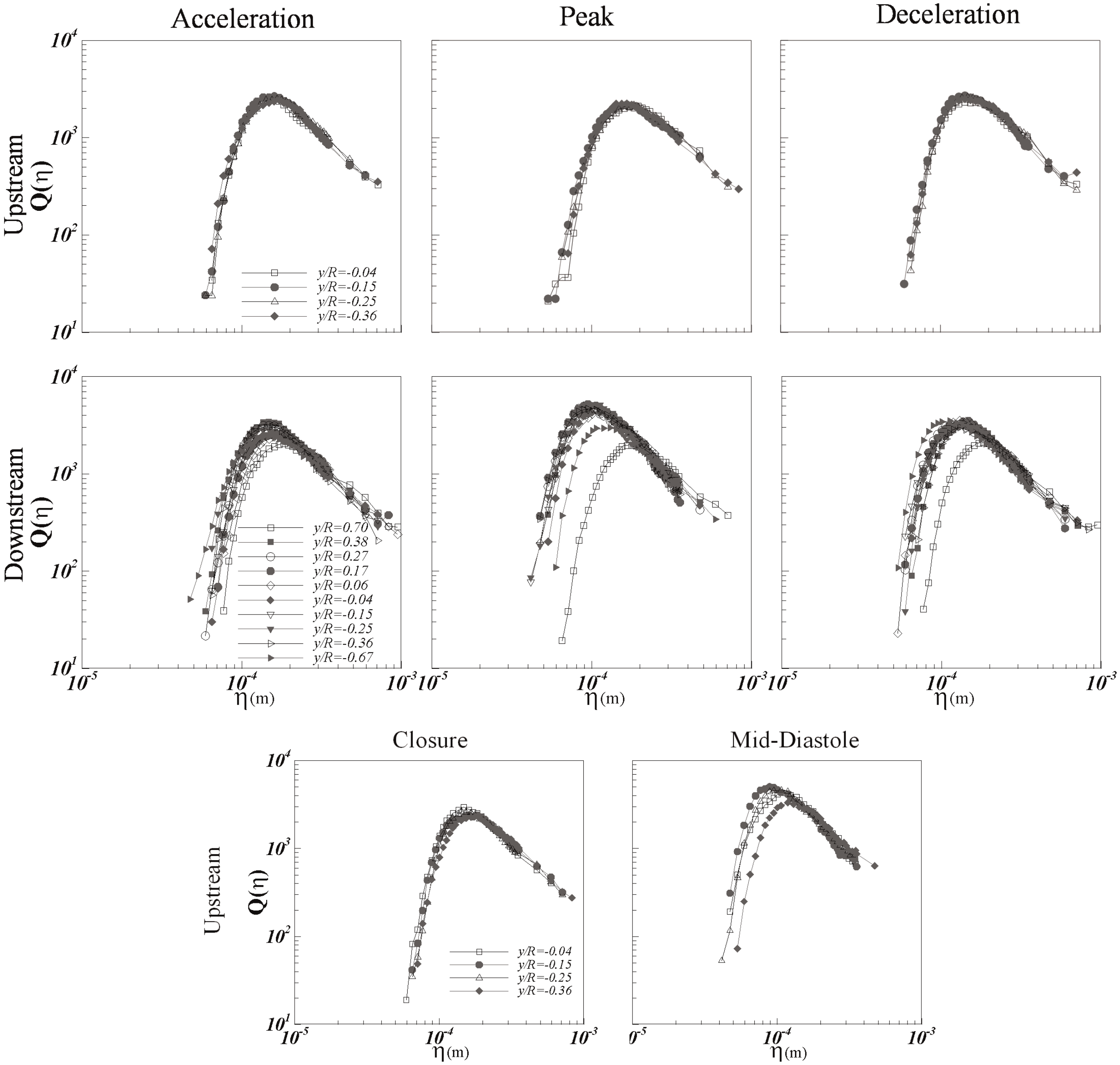

Figure 4. Probability density function Q(η) during acceleration, peak, and deceleration phases at the points of interest upstream (top row) and downstream (middle row). Closure and mid-diastole phases for points of interest upstream are shown in the bottom row.
doi:10.1371/journal.pone.0105357.g004

stress acting on RBCs and is perhaps insufficient to reflect the intense fluctuations of the dissipation rate that would correspond to sub-Kolmogorov scale eddies. Thus, to fully capture the statistical nature of shear stress acting on RBC membranes, it is necessary to characterize the probability density function of τ_p denoted $P(\tau_p)$. $P(\tau_p)$ is related to the probability density function of η, $Q(\eta)$, through conservation of probability as: $P(\tau_p)=Q(\eta)\frac{d\eta}{d\tau_p}$. Recent turbulence literature indicates that $Q(\eta)$ may be universal despite the highly intermittent fluctuations of the instantaneous dissipation rate field [18,19]. Analytical forms for this universal distribution exist with good agreement for experiments and simulations [24,27]. The key point relevant here is that $P(\tau_p)$may be universal in the strongly turbulent regions of blood flow through devices, and estimated using a simple change of variables as:

$$P(\tau_p)=-\frac{1}{2}C^{1/2}\tau_p^{-3/2}Q\left(\frac{L\tau_p^{1/2}}{C^{1/2}}\right) \qquad (3)$$

Where $C=v_B\sqrt{\frac{\mu_B\mu_p}{(1-H)}}$.

The above arguments and physical construction not only provides a mathematical relationship between η(as a surrogate for ε), and τ_p, but also yields $P(\tau_p)$. However, how does this relationship link turbulence statistics to τ_p? The most common

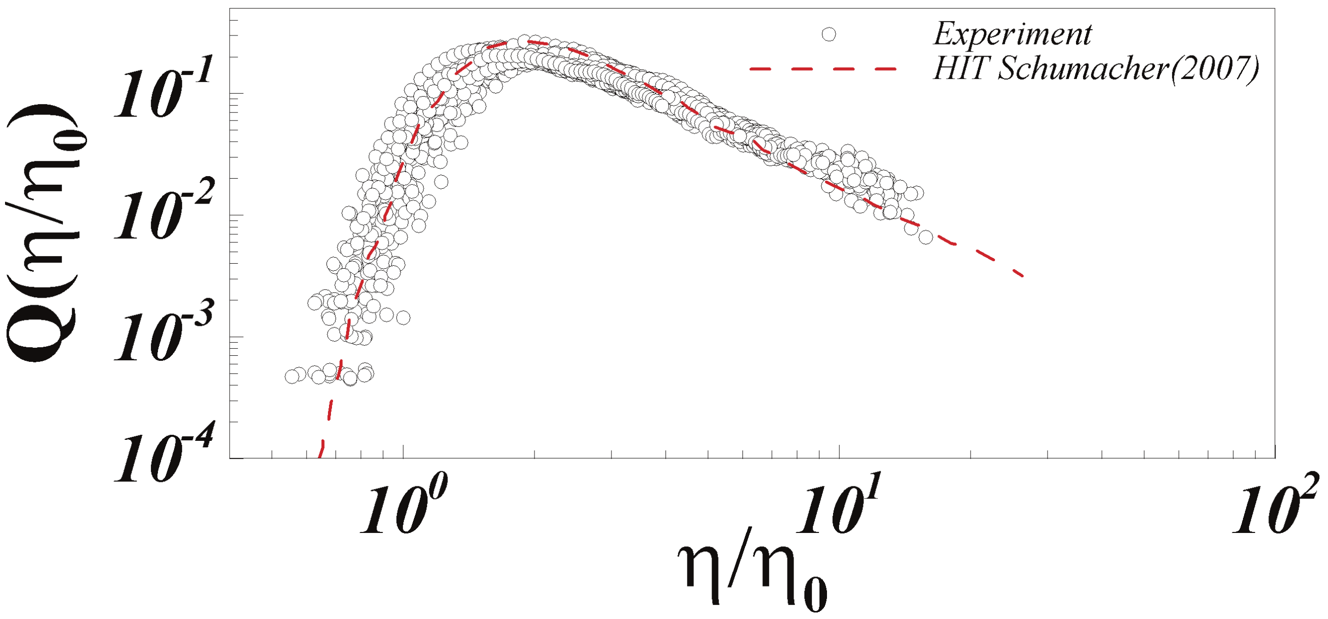

Figure 5. Normalized probability density function $Q(\eta/\eta_0)$ at the points of interest upstream and downstream at all phases compared to published isotropic result.
doi:10.1371/journal.pone.0105357.g005

and perhaps paradoxical turbulence statistic in the context of blood damage has been the Reynolds stress tensor $\rho\langle u_i u_j\rangle$. This has been extensively discussed in the past (and key issues reviewed in [12]) with the note that while it does appear to relate to predicting cell damage, there is no consensus with respect to the physical relationship between $\rho\langle u_i u_j\rangle$ and τ_p. An alternate model to estimate τ_p based on cell relative velocities between adjacent eddies has been proposed [12] but in our opinion this lacks the physical justification for existence of such intense velocity jumps (more severe than the smallest sub-Kolmogorov event) over sub-micron scales. Nevertheless, based on the phenomenological arguments made earlier with the hypothetical eddy, it is easy to see that it is the total energy dissipation rate that directly dictates τ_p. One feasible explanation we can offer to explain the robustness of Reynolds shear stress in predicting blood damage is that for regions in equilibrium, the average rate of energy dissipation, ε is related to Reynolds stress given by the equation (proof in [34]): $\epsilon \approx -\langle u_i u_j\rangle \frac{dU_i}{dx_j}$, where $\frac{dU_i}{dx_j}$ is the mean strain rate tensor. Plugging this approximation in equation 1 and ensemble averaging it, the root mean square of τ_p is given by:

$$\langle \tau_p^2\rangle^{1/2} \approx \left[-\frac{\mu_p \rho_B}{1-H}\langle u_i u_j\rangle \frac{dU_i}{dx_j}\right]^{1/2} \quad (4)$$

The above discussion is presented for completeness and offers to reconcile the rather paradoxical issue (so far) of why Reynolds shear stress has been effective in capturing blood damage potential of turbulent blood flows [12]. The reconciliation that we offer through equation 4 is that it is not the Reynolds stress itself, but the product of the Reynolds stress and the local strain rate that determines the energy passed on to the cascade and hence the total energy dissipation rate, which as we have shown should ultimately set the viscous shear stress acting on blood cell membranes. A rough order of magnitude verification of equation 4 can be made based on the contour scale bars in Ref. [14] that experimentally quantifies the Reynolds shear stresses in a heart valve flow. By setting Reynolds shear stress in equation 4~100 N/m^2 (i.e. O(100) N/m^2), and substituting μ_p to be $\sim 10^{-3}$ Ns/m^2 and mean strain rate to be O(1000) s^{-1}, we get $\langle \tau_p^2\rangle^{1/2} \sim 14 N/m^2$. This strikingly agrees with the range of viscous shear stress reported in Ref. [14] to be 15 N/m^2. This agreement demonstrates further that Reynolds shear stress when combined with the mean strain rate can relate as an order of magnitude estimate to viscous shear stress acting on blood cells. The most comprehensive representation of shear stress acting on cells however is undoubtedly the hypothesized universal distribution function $P(\tau_p)$.

A Test of Universality and Quantification of $P(\tau_p)$. With the reasonableness of the Newtonian assumption for physiological turbulent eddies, we interrogate the most complex turbulent blood flow problem — the pulsatile turbulent field surrounding a bileaflet mechanical heart valve using high-resolution phase-locked particle image velocimetry to calculate $P(\tau_p)$ and assess its universality. Turbulence in mechanical heart valve flows has received enormous attention in medical research with unresolved issues in relation to the applicability of Reynolds shear stresses on blood cells [12]. Briefly, a bileaflet mechanical heart valve was experimentally subjected to physiological aortic conditions and the instantaneous turbulent velocity field was captured in the vicinity of the valve during representative phases of the cardiac cycle. Points of interest within the measurement region where complexity is expected (Figure 3) were further interrogated to quantify the probability density function $Q(\eta)$ and the corresponding probability density function of the shear stress, $P(\tau_p)$ for a hematocrit of 48%.

Particle Image Velocimetry (PIV) of Bileaflet Mechanical Heart Valve. Experiments are similar to that described in Ref. [35]. The fluid was seeded with polyamide tracer particles (Dantec Dynamics, Denmark) distributed in the 5 to 30 microns range. A laser sheet illuminated the central plane normal to the b-datum line. The laser was generated using the Photonics Industries DM40-527 diode-pump Q-switched laser (Photonics, Bohemia,

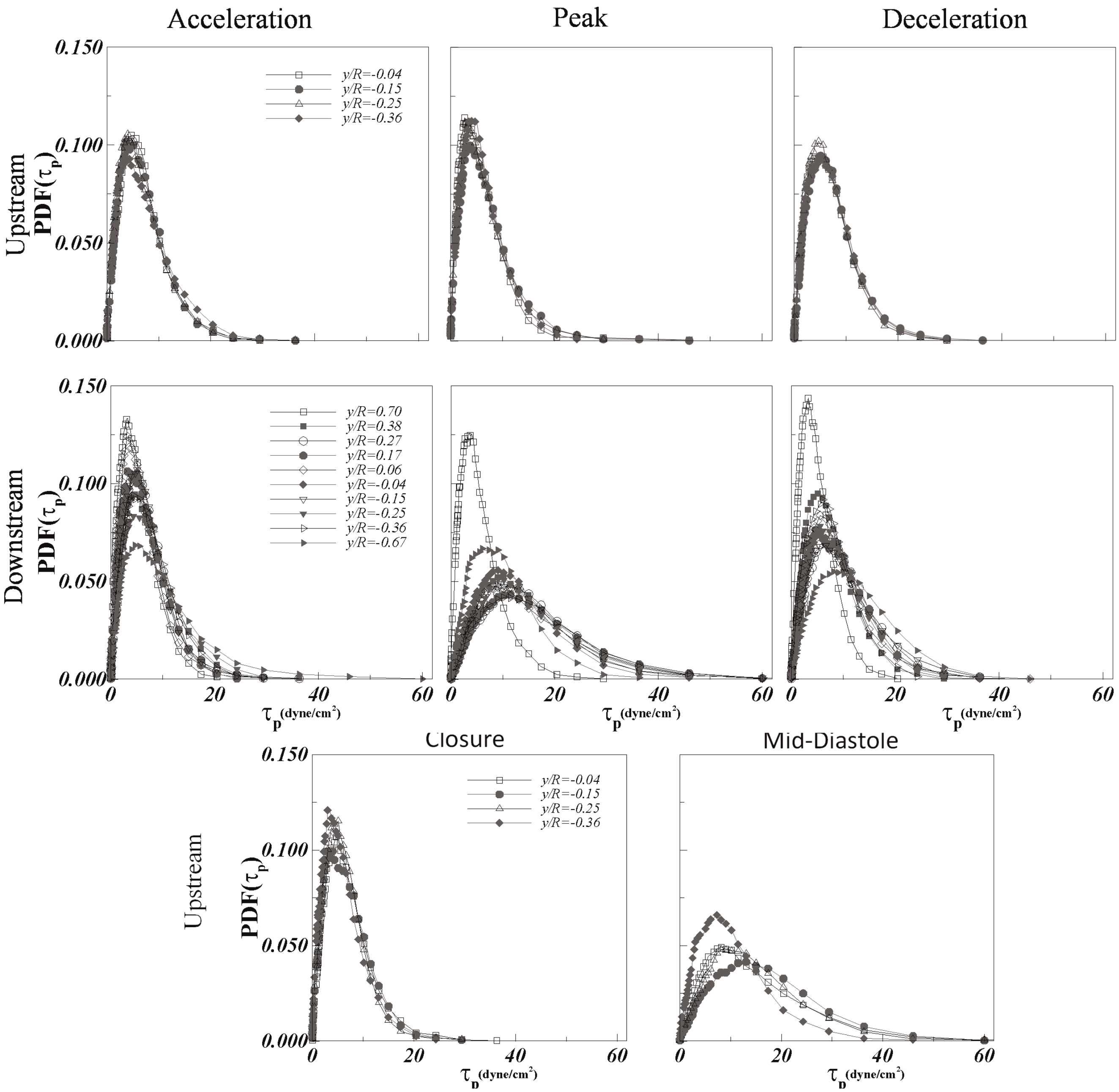

Figure 6. Probability density function of instantaneous shear stress (τ_p) during acceleration, peak, and deceleration phases at the points of interest upstream (top row) and downstream (middle row). Closure and mid-diastole phases for points of interest upstream are shown in the bottom row.
doi:10.1371/journal.pone.0105357.g006

NY) with optics to covert the output beam into an expanded laser sheet. The laser had an initial thickness of approximately 1 mm, which was focused down to less than 200 microns within the measurement region using a spherical lens ($f = 1$ m). The valve was oriented such that the measurement plane bisected both leaflets at the central plane of valve model. A Photron Fastcam SA3 CCD high speed video camera (Photron, San Diego, CA) synchronized to the laser system via a high speed controller (HSC) (LaVision, Ypsilanti, MI) captured focused images of the illuminated polyamide particles within the laser sheet in the measurement plane. The image area of interest was 1.5D wide and 1D tall with the valve body centered. Image distortion due to curvature of the acrylic tube was compensated in-situ with a calibration plate consisting markers placed in a regular square grid with 1 mm spacing. The DaVis calibration algorithm (LaVision Inc, Germany) automatically tracks the markers and a map to evaluate the corrected image. Corrected image generated of the calibration plate verified successful calibration and distortion correction. The PIV setup achieved a raw data spatial resolution of roughly 27 μm/pixel. PIV measurements were performed in double-frame mode with a laser pulse separation time, $\Delta t = 500$ μs. This ensured adequate particle displacements in the range of 10–15 pixels thus maximizing the accuracy of instantaneous velocity measurements to within 2% error.

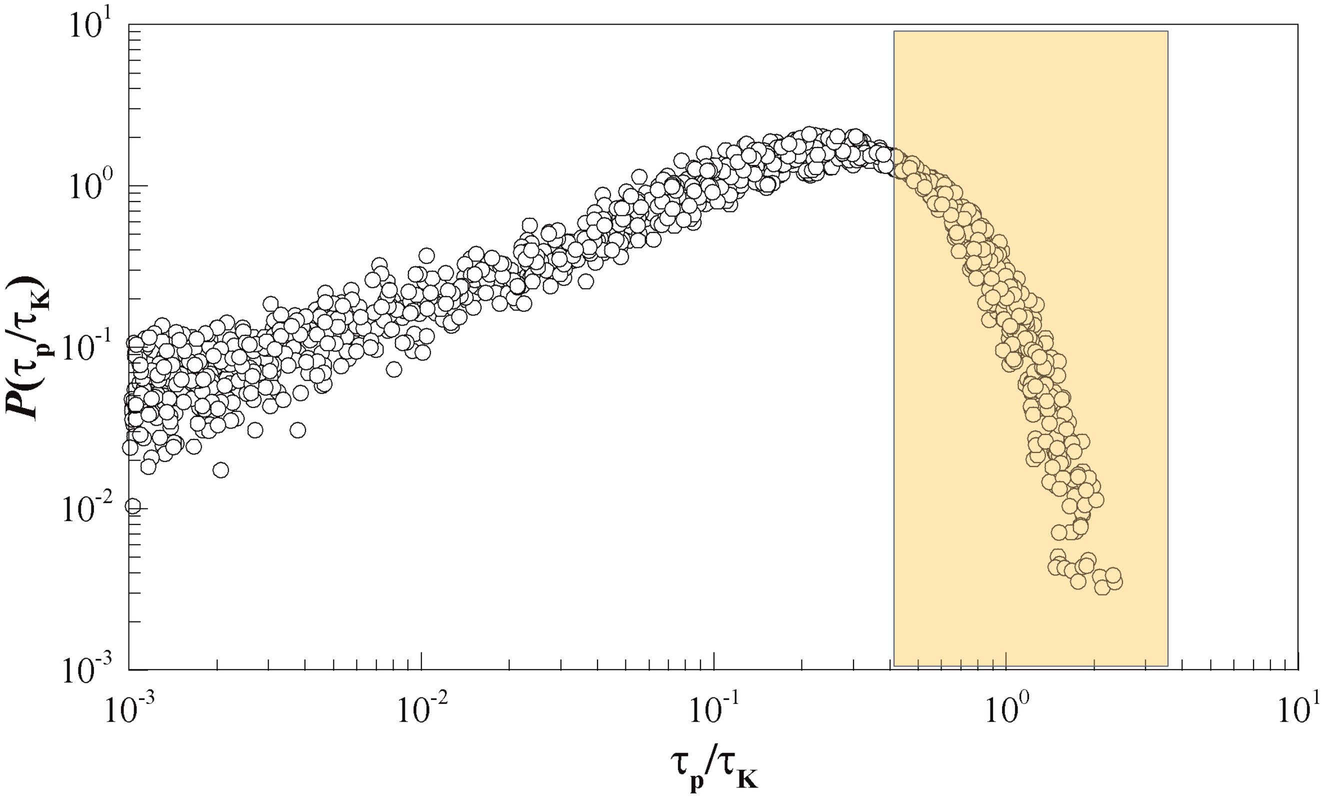

Figure 7. Probability density function of instantaneous normalized shear stress (τ_p/τ_K) for all points of interest at all phases. Notice the close data collapse for τ_p/τ_K above 0.5. The probability of $\tau_p > 0.5\tau_K$ is about 0.36 (shaded region).
doi:10.1371/journal.pone.0105357.g007

Images were pre-conditioned by first subtracting the minimum image from every image acquired. Instantaneous two-dimensional velocity field was calculated from the raw particle images using cross-correlation processing with a multi-pass scheme. The initial interrogation window size for the multi-pass scheme was at 32×32 pixels, which progressively reduced to 8×8 pixels. Interrogation window overlap was fixed at 50%. Post-processing of the vector data included a median filter that rejected vectors outside 3 standard deviations of the neighbor vector. Gaussian smoothing was used to reduce noise in the vector data. An in-house Matlab code was developed to post-process these raw velocity measurements to derive statistical properties, specifically PDFs.

Peak locking index was calculated to be between 0.02–0.19. Peak locking index is defined as $4|\phi - \frac{1}{4}|$ where ϕ is the first moment of the probability distribution function of the absolute fractional distance in pixels to the nearest integer pixel displacement. If the probability distribution is uniform in the pixel displacement range 0 to 0.5, then the pixel locking index is zero, indicating no pixel locking. A value of 1 indicates 100% pixel locking (i.e. no sub-pixel displacements recorded). Our range of 0.02–0.19 for peak locking index is far below 0.25, which is the threshold for minor peak locking artifacts.

Error Considerations. The sources of error in our measurements are due to resolution as well as random error. Random errors are addressed in this study through statistical averaging of repeated measurements (N = 500) and statistical comparisons. This section briefly outlines the errors in accuracy due to limited resolution of the measurement techniques at hand. The conservative error estimate in velocity is <2% (i.e. particle displacements may be off by ±0.2 pixels out of total displacement of 10 pixels). Laser pulse timing errors are negligible in comparison.

Validation. In order to check if the valve chamber and the acrylic model valve provide equivalent results to clinical quality St. Jude Medical valve, Figure 5 of Ref. [35] compares non-dimensionalized leaflet kinematics, and the downstream velocity profile at $x/D = 0.33$ during peak systole to published results for a clinical quality St. Jude Medical valve [36].

Calculation of $Q(\eta)$ and $P(\tau_p)$. PDFs of the local dissipation scale, $Q(\eta)$ was calculated for each interrogation point in Figure 3. The approach is similar to that previously described [20,37] with the qualifying condition for η being $0.9 < \frac{\eta \delta u_\eta}{\nu} < 2$. Briefly, the instantaneous η is estimated from the local velocity increment between PIV velocity measurement points. We have shown in Ref. [37] that a significant portion of the local dissipative scale PDF, $Q(\eta)$ may be derived if the velocity measurement resolves a sizeable portion of the dissipative range; i.e. PIV resolution well resolves the Taylor microscale. Given that the PDF is derived from the histogram of the occurrence of η, and that the measured variable in the above inequality is δu_η, it is straight forward to propagate the percent error in the instantaneous velocity of the PIV measurements to the uncertainty in the PDF. Our uncertainty of 2% in velocity translates to an uncertainty of 2.8% in δu_η. Given that the inequality is the only qualifying criteria, the uncertainty in the PDF may be achieved by perturbing the upper and lower limits of the inequality. To be conservative, we recalculated the PDFs by incorporating a 10% variation in the limits and found that the resulting PDFs with this additional 10% uncertainty in δu_ηinsignificantly influenced the shape of the PDF.

Table 3. Mean and minimum dissipative eddies observed along with corresponding shear stress magnitudes – Upstream.

	y/R	η_{min} (μm)	τ_{max} Current Study (dyne/cm^2)	η_{avg} (μm)	$\tau_{\eta avg}$ Current Study (dyne/cm^2)	η_{peak} (μm)	$\tau_{\eta peak}$ Current Study (dyne/cm^2)
**Acceleration**	−0.04	75	18.61	273	1.39	145	4.94
	−0.15	73	19.17	261	1.52	158	4.14
	−0.25	77	17.25	268	1.44	161	3.98
	−0.36	70	21.35	268	1.44	166	3.75
**Peak**	−0.04	83	15.10	288	1.25	173	3.45
	−0.15	78	17.00	285	1.28	164	3.86
	−0.25	78	17.09	290	1.23	181	3.15
	−0.36	75	18.31	284	1.28	155	4.32
**Deceleration**	−0.04	73	19.33	274	1.38	146	4.83
	−0.15	69	21.90	258	1.55	137	5.53
	−0.25	76	17.99	264	1.48	146	4.83
	−0.36	79	16.38	259	1.54	146	4.83
**Regurgitation**	−0.04	70	21.11	251	1.64	146	4.83
	−0.15	61	27.56	264	1.48	162	3.95
	−0.25	69	21.58	266	1.46	145	4.94
	−0.36	67	22.76	279	1.33	175	3.37
**Diastole**	−0.04	57	31.32	193	2.77	110	8.49
	−0.15	49	42.86	178	3.26	91	12.44
	−0.25	57	31.57	191	2.84	108	8.88
	−0.36	55	34.70	219	2.16	119	7.25

doi:10.1371/journal.pone.0105357.t003

Table 4. Mean and minimum dissipative eddies observed along with corresponding shear stress magnitudes – Downstream.

	y/R	η_{min} (μm)	τ_{max} Current Study (dyne/cm^2)	η_{avg} (μm)	$\tau_{\eta avg}$ Current Study (dyne/cm^2)	η_{peak} (μm)	$\tau_{\eta peak}$ Current Study (dyne/cm^2)
**Acceleration**	0.70	94	11.60	641	0.25	175	3.37
	0.38	62	26.61	298	1.17	146	4.83
	0.27	68	22.65	352	0.83	146	4.83
	0.17	81	15.94	433	0.55	171	3.53
	0.06	80	16.24	504	0.41	173	3.45
	−0.04	83	15.17	434	0.55	141	5.17
	−0.15	70	21.32	380	0.72	140	5.29
	−0.25	75	18.44	334	0.93	132	5.92
	−0.36	63	25.85	380	0.72	141	5.17
	−0.67	57	32.31	373	0.74	138	5.41
**Peak**	0.70	87	13.66	689	0.22	169	3.61
	0.38	45	50.27	158	4.12	100	10.39
	0.27	45	51.13	156	4.25	92	12.16
	0.17	45	51.45	151	4.53	95	11.37
	0.06	51	39.41	188	2.93	110	8.49
	−0.04	49	42.87	185	3.02	108	8.88
	−0.15	45	50.35	155	4.28	101	10.16
	−0.25	43	56.25	170	3.57	104	9.50
	−0.36	41	62.58	164	3.82	96	11.12
	−0.67	62	26.68	310	1.07	128	6.33
**Deceleration**	0.70	99	10.57	690	0.22	175	3.37
	0.38	67	22.85	336	0.92	143	5.06
	0.27	72	20.07	271	1.40	126	6.48
	0.17	61	28.17	277	1.35	143	5.06
	0.06	68	22.36	271	1.40	131	6.05
	−0.04	66	23.76	284	1.28	131	6.05
	−0.15	65	24.55	274	1.37	129	6.19
	−0.25	63	26.07	297	1.17	132	5.92
	−0.36	69	21.95	333	0.93	131	6.05
	−0.67	68	22.15	233	1.90	110	8.49
**Regurgitation**	0.70	62	26.75	691	0.22	185	3.01
	0.38	145	4.90	633	0.26	183	3.08
	0.27	143	5.07	499	0.42	160	4.04
	0.17	158	4.16	630	0.26	176	3.34
	0.06	143	5.06	544	0.35	183	3.08

Table 4. Cont.

	y/R	η_{min} (μm)	τ_{max} Current Study (dyne/cm^2)	η_{avg} (μm)	$\tau_{\eta avg}$ Current Study (dyne/cm^2)	η_{peak} (μm)	$\tau_{\eta peak}$ Current Study (dyne/cm^2)
	−0.04	122	6.90	457	0.49	169	3.61
	−0.15	179	3.24	541	0.35	156	4.22
	−0.25	152	4.46	527	0.37	167	3.69
	−0.36	153	4.39	494	0.42	146	4.83
	−0.67	138	5.43	878	0.13	194	2.75
**Diastole**	0.70	94	11.80	740	0.19	175	3.38
	0.38	89	13.04	663	0.23	175	3.37
	0.27	88	13.38	612	0.28	158	4.13
	0.17	91	12.44	636	0.26	179	3.23
	0.06	93	11.90	723	0.20	173	3.45
	−0.04	90	12.68	709	0.21	160	4.04
	−0.15	98	10.69	710	0.21	177	3.30
	−0.25	100	10.38	746	0.19	173	3.45
	−0.36	99	10.65	767	0.18	169	3.61
	−0.67	108	8.86	969	0.11	207	2.41

doi:10.1371/journal.pone.0105357.t004

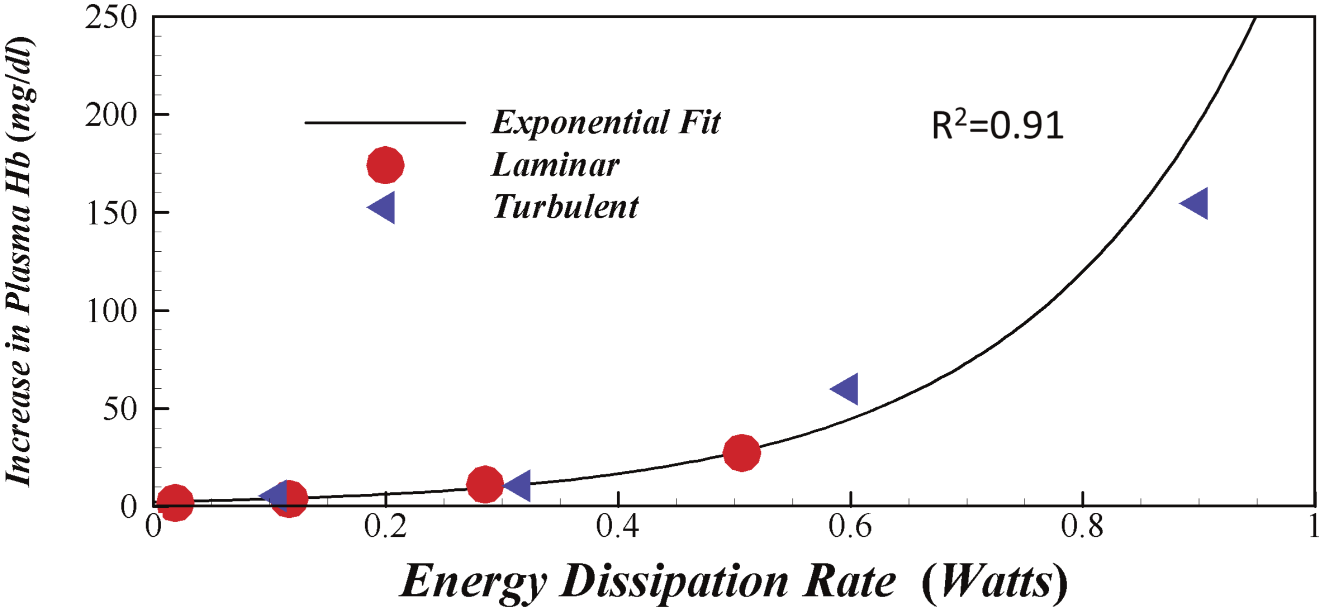

Figure 8. A plot of plasma hemoglobin data from Kameneva et. al (2009) with respect to total energy dissipation rate calculated from reported wall shear stress and Reynolds numbers. Figure illustrates how total energy dissipation consistently captures blood damage irrespective of laminar or turbulent flow regimes.
doi:10.1371/journal.pone.0105357.g008

The PDF $P(\tau_p)$ was determined by directly calculating the occurrences of τ_p for every occurrence of η using equation 2. Non-dimensionalization parameters were calculated for each interrogation point and are listed in Tables 1 and 2.

Results: Dimensional Pdfs of η and $\tau_p$for Flow Near a Bileaflet Mechanical Heart Valve

Figure 4 presents the probability density function $Q(\eta)$ at points of interest (defined in Figure 3) during specific phases of the cardiac cycle. Upstream of the valve, the peak of $Q(\eta)$ (representing the mode of the fluctuating η) is consistently observed to be between 100–200 μm during forward flow. The smallest eddies captured in the distribution function are in the range 50–70 μm. The same characteristics are observed upstream of the valve during closure phase. However, during mid-diastole, there is a significant change in $Q(\eta)$ with respect to the varying location of points. In particular, except for the furthest location from the centerline, the locations closer to the centerline correspond to a significant leftward shift of $Q(\eta)$. For these locations, the mode of η is in the range 80–90 μm, with the smallest η about 40 μm. Downstream of the valve, there is significant variation in $Q(\eta)$ characteristics as a function of the lateral position relative to the centerline during acceleration, peak, and deceleration phases (Figure 4). The smallest η is between 40–50 μm and the mode of η between 90–200 μm. Positions located within the side orifice jets above and below the centerline do not show significantly different $Q(\eta)$ characteristics compared to the upstream forward flow characteristics.

The above shifts in $Q(\eta)$ characteristics clearly point to the slight leftward shift whenever increased turbulence is expected. For instance, locations slightly off from the centerline upstream of the valve will experience the high shear regions of the regurgitation jet during mid-diastole. Similarly, the leftward shifting of $Q(\eta)$, also coincides with the shear layer locations downstream of the two leaflets consistent with the results in a more classical flow problem [36]. For all the locations, it appears that the smallest eddies are in the neighborhood of about 40 μm, which is consistent with the literature [12]. On the contrary, these small eddies appear to be relatively rare events with most of the dissipative eddies in the turbulent zones around 80 μm. This may be lower in locations where significantly greater turbulence exists.

Figure 5 presents the normalized probability density function $Q(\eta/\eta_O)$ for each of the positions of interest upstream and downstream at different phases of the cardiac cycle. η_O is defined as $\eta_O = LRe_L^{-0.72}$ where L is the local integral length scale, and $Re_L = k^{1/2}L/\nu$ as discussed in Refs. [18,26] is a scale very close to $\eta_K = LRe_L^{-3/4}$. Also presented is $Q(\eta/\eta_O)$ for the case of homogenous isotropic turbulence (HIT) from highly resolved direct numerical simulations [26] for comparison. Figure 5 shows very good agreement of the experimentally derived PDFs with the HIT expectation. The scatter around the HIT expectation may be attributed to experimental uncertainty as well as weak dependence of $Q(\eta/\eta_O)$ on local mean shearing [20]. Specifically, we have shown that this weak dependence occurs when the Corrsin length scale, $\eta_c = \left(\langle\epsilon\rangle/S^3\right)^{1/2}$, where S is the mean shear magnitude, approaches the mean shear-viscosity length scale, $(\nu/S)^{1/2}$ [20]. Nevertheless, from a practical standpoint these results confirm the largely valid assumption of universal small scale structures despite the highly pulsatile nature of the turbulent flow past a complex device. This is particularly significant given the classical assumptions behind universality of turbulence are highly restrictive (i.e. very high Reynolds number, fully developed, equilibrium, stationary etc.).

Figure 6 presents the raw PDFs of τ_p. The peak (mode) of the distribution for τ_p ranges between 5 and 20 dynes/cm^2 with the right tail extending to about 60 dynes/cm^2. Tables 3 and 4 list the maximum observed τ_p corresponding to the minimum η at each position for all recorded phases for the cardiac cycle. To assess the universality of $P(\tau_p)$, we examine the normalized probability density function $P(\tau_p/\tau_{p_K})$ (see Figure 7) for the different points of

interest and cardiac phases. τ_{p_K} is defined by substituting $\eta_K = LRe_L^{-3/4}$ in equation 2, thus defining a characteristic Kolmogorov scale shear stress acting on blood cells as:

$$\tau_{pK} = \sqrt{\frac{\mu_B \mu_p}{1-H}} \frac{k^{1/2}}{L} {Re_L}^{1/2} \tag{5}$$

The normalized PDFs show strong data collapse indicating a practical universality which is expected based on the collapse observed for $Q(\eta/\eta_O)$. Note that the maximum observable τ_p was roughly $2\tau_{p_K}$ regardless of location of measurement or cardiac phase for the specific problem. The tale of $P(\tau_p/\tau_{p_K})$ drops exponentially around this magnitude. A theoretical limit to the maximum shear stress $\tau_{p_{max}}$ may be estimated utilizing the smallest possible sub-Kolmogorov scale $\eta_S = L{Re_L}^{-1}$ [26,28] to yield $\tau_{p_{max}} = \sqrt{\frac{\mu_B \mu_p}{1-H}} \frac{k^{1/2}}{L} Re_L$.

Discussion

Resolving Historically Inconsistent Hemolysis in Laminar and Turbulent Pipe Flow

The argument introduced to relate turbulence properties to the shear stress environment is a simple balance of energy dissipation. Thus, regardless of whether a flow is laminar or turbulent, isotropic or not, energy dissipated must highly correlate to the fate of suspended cells. In fact, energy dissipation was classically recognized as the hemodynamic parameter that dictates hemolysis based on simple physical arguments [38,39]. We test if this can resolve one of the paradoxical results published in Ref. [13] where hemolysis was measured in fully developed laminar and turbulent pipe flows while maintaining the same wall shear stress. Given that the total shear stress of the mean flows for both these cases are identical, the conventional thinking expects the same levels of hemolysis. However, turbulent cases produced significantly higher hemolysis [13]. To resolve the lack of an appropriate physical interpretation, we present a revised graph (Figure 8) that plots the measured plasma free hemoglobin (a measure hemolysis) as a function of total energy dissipation rate (in watts) in the pipe based on available information of wall shear stress and Reynolds numbers. Briefly, based on the wall shear stress magnitudes, length of the pipe, and its diameter the pressure drop can be calculated using a simple force balance. We also calculated flow rates from the given Reynolds number, viscosity, and pipe diameter information. The pressure drop and flow rate were multiplied to yield the total energy dissipation rate in watts. Figure 8 confirms that indeed, regardless of whether the flow is laminar or turbulent, the total energy dissipated produces a single monotonic functional dependence for hemolysis. The functional form with the best correlation was an exponential fit with $R^2 = 0.91$ compared to linear, and power fit models. Nonetheless, this result supports our fundamental assumptions and confirms that total energy dissipation is the fundamental parameter that dictates the mechanical environment of suspended cells.

One must note however that in the above pipe flow data, exposure time was not a relevant quantity as in all the experiments of ref. [13], the total exposure time was fixed. Thus the resulting trend shown in Figure 8 reflects the exponential growth in hemolysis due to the complex instantaneous energy dissipation "histories" of the turbulent cases. Further studies are necessary to develop appropriate blood damage models that take into account instantaneous energy dissipation rate histories and exposure times for pulsatile applications.

In the Context of a Cellular Environment

Cells are highly responsive to changes in their energetic environment, as demonstrated by the effectiveness of *in vivo* laser injury models [40]. Thermal energy is capable of inducing alterations to RBC morphology and can result in hemolysis in extreme cases [41,42]. Exacerbating the problem, a cascade of events can occur upon RBC lysis including platelet activation stimulated from adenosine diphosphate [43]. More direct studies of mechanical stress have demonstrated numerous effects on suspended blood cells, which can be combined with an energy balance equation, as presented here to determine the degree of cell activity. High shear stress is known to cause hemolysis and can further result in shear-induced platelet activation and shear-induced platelet aggregation, which has become known as SIPA, a feature that occurs independent of activation agonists. This process depends on the magnitude and the duration of shear stress [33]. More complex gradients in shear stress have been shown to promote activation-independent platelet aggregation involving the binding protein, von Willebrand factor (VWF) [44], a feature that may be important for the intermittent nature of turbulence. Platelets combined with VWF can result in aggregation while in suspension, independent of an adhesive surface substrate [45]. For sufficient shear stress, VWF can change from a globular confirmation to an extended chain [46,47], which can result in self-association, creating a structure that is very adherent to platelets and can create a physical barrier for other cells [48]. Shear stress in bulk flow or at a surface will also influence the inflammatory response. Activated platelets, alone, release a number of prothrombotic and proinflammatory molecules through α-granules and microparticles [49–51]. These processes may interact with mechanotransductive mechanisms in monocytes, which are highly responsive to membrane tension through cellular activation, signaling, and migration [52,53].

The current model may better predict the local shear stress environment for these processes and for dictating the function of binding proteins, platelets, RBCs, and monocytes, especially for the complex flow environment that can exist in medical devices and atherosclerosis [54]. As it becomes clear how cells respond to their mechanical environment, it will be increasingly important to develop unifying theories, such as the one presented in this paper to better determine the mechanical conditions for cells in a physiological or pathophysiological environment. It will further be critical for medical device development to consider the relationship between the cellular response and mechanics. For example, it has already been shown that self-association of VWF can result in acquired von Willebrand Disease in devices, including ventricular assist devices [55,56], a blood disorder that can be amplified by shear-induced receptor shedding in platelets [57].

In addition to predicting cellular functional responses to the local mechanical environment, it is also important to consider the influence on transport in the fluid flow. The complex biorheology of blood is highly dependent on the local shear environment [58]. The local environment influences the number of collisions between cells and the rotational nature of the cells, both of which are largely dependent on the hematocrit. Transport in the flow may be critical for thrombus growth rates and the movement of microparticles or cellular agonists [59,60].

Limitations

The proposed theory assumes that the distribution of eddies are not largely influenced by factors, such as the mean shear in the

flow environment, an assumption shown to be reasonably valid for the mean shear at the points examined. However, there may be much stronger shearing in medical devices such as LVADs, where strong mean shear effects need to be accounted for to revise the distribution function of the dissipative scales of equation 3. In our probability density functions, we did see slight shifts, which may be caused by these influences, discussed in detail elsewhere [20,37]. However, the shifts were relatively minor in the current study.

Conclusion

We introduced a theory to predict the mechanical environment of cells in turbulent blood flows through physical arguments applicable to any in-homogenous turbulent flow. It is argued that for the case of physiological blood flow, the dissipative turbulent eddies are well in the Newtonian regime of blood rheology. We experimentally showed that the "universal" prediction of dissipative eddy distributions is valid even in a highly complex flow generated past a bileaflet mechanical heart valve, and that consequently the shear stress distributions experienced by blood cells must follow a practically universal distribution function. The universal form for shear stress acting on cells is presented and the theoretical maximum shear stress in a turbulent flow defined. Finally, the underlying assumption that local energy dissipation rate is the key to predicting the stress environment of cells is tested by demonstrating the high correlation between hemolysis and energy dissipation in pipe flow regardless of laminar or turbulent flow regime.

Acknowledgments

The authors gratefully acknowledge funding from National Institutes of Health (NIH) under Award Number R01HL119824, and the American Heart Association under award 11SDG5170011. The content is solely the responsibility of the authors and does not necessarily represent the official views of the NIH.

Author Contributions

Conceived and designed the experiments: LD. Performed the experiments: MF. Analyzed the data: LD KM. Wrote the paper: LD KM DB.

References

1. Coulter NA, Pappenheimer JR (1949) "DEVELOPMENT OF TURBULENCE IN FLOWING BLOOD," American Journal of Physiology, 159(2), pp. 401–408.
2. Ku DN (1997) "Blood flow in arteries," Annual Review of Fluid Mechanics, 29, pp. 399–434.
3. Bramwell ST, Holdsworth PCW, Pinton JF (1998) "Universality of rare fluctuations in turbulence and critical phenomena," Nature, 396(6711), pp. 552–554.
4. Nelkin M (1992) "IN WHAT SENSE IS TURBULENCE AN UNSOLVED PROBLEM," Science, 255(5044), pp. 566–570.
5. Sreenivasan KR (1991) "FRACTALS AND MULTIFRACTALS IN FLUID TURBULENCE," Annual Review of Fluid Mechanics, 23, pp. 539–+.
6. Sreenivasan KR, Antonia RA (1997) "The phenomenology of small-scale turbulence," Annual Review of Fluid Mechanics, 29, pp. 435–472.
7. Nicklin MJH, Hughes DE, Barton JL, Ure JM, Duff GW (2000) "Arterial inflammation in mice lacking the interleukin 1 receptor antagonist gene," J. Exp. Med., 191(2), pp. 303–311.
8. Beckman JS, Koppenol WH (1996) "Nitric oxide, superoxide, and peroxynitrite: The good, the bad, and the ugly," Am. J. Physiol.-Cell Physiol., 271(5), pp. C1424–C1437.
9. Tay JH, Liu QS, Liu Y (2001) "The effects of shear force on the formation, structure and metabolism of aerobic granules," Appl. Microbiol. Biotechnol., 57(1-2), pp. 227–233.
10. Bark Jr DL, Nesbitt WS, Wong AKT, Jackson SP (2013) "Shear Effects on Platelets and the Vessel Wall in the Pathogenesisi of Atherothrombosis," Hemostasis and Thrombosis: Basic Principles and Clinical Practice, Lippincott Williams & Wilkins, p. 1592.
11. Grigioni M, Daniele C, D'Avenio G, Barbaro V (1999) "A discussion on the threshold limit for hemolysis related to Reynolds shear stress," J. Biomech., 32(10), pp. 1107–1112.
12. Antiga, L., and Steinman, D. A., 2009, "Rethinking turbulence in blood," Biorheology, 46(2), pp. 77–81.
13. Kameneva MV, Burgreen GW, Kono K, Repko B, Antaki JF, Umezu M (2004) "Effects of turbulent stresses upon mechanical hemolysis: Experimental and computational analysis," ASAIO J., 50(5), pp. 418–423.
14. Ge L, Dasi LP, Sotiropoulos F, Yoganathan AP (2008) "Characterization of hemodynamic forces induced by mechanical heart valves: Reynolds vs. viscous stresses," Annals of Biomedical Engineering, 36(2), pp. 276–297.
15. Bellofiore A, Quinlan NJ (2011) "High-Resolution Measurement of the Unsteady Velocity Field to Evaluate Blood Damage Induced by a Mechanical Heart Valve," Annals of Biomedical Engineering, 39(9), pp. 2417–2429.
16. Dooley PN, Quinlan NJ (2009) "Effect of Eddy Length Scale on Mechanical Loading of Blood Cells in Turbulent Flow," Annals of Biomedical Engineering, 37(12), pp. 2449–2458.
17. Quinlan NJ, Dooley PN (2007) "Models of flow-induced loading on blood cells in laminar and turbulent flow, with application to cardiovascular device flow," Annals of Biomedical Engineering, 35(8), pp. 1347–1356.
18. Bailey SCC, Hultmark M, Schumacher J, Yakhot V, Smits AJ (2009) "Measurement of Local Dissipation Scales in Turbulent Pipe Flow," Phys. Rev. Lett., 103(1).
19. Hamlington PE, Krasnov D, Boeck T, Schumacher J (2012) "Local dissipation scales and energy dissipation-rate moments in channel flow," Journal of Fluid Mechanics, 701, pp. 419–429.
20. Morshed KN, Venayagamoorthy SK, Dasi LP (2013) "Intermittency and local dissipation scales under strong mean shear," Physics of Fluids, 25(1).
21. Yun BM, Dasi LP, Aidun CK, Yoganathan AP (2014) "Highly resolved pulsatile flows through prosthetic heart valves using the entropic lattice-Boltzmann method," Journal of Fluid Mechanics, 754, pp. 122–160.
22. Fahraeus R, Lindqvist T (1931) "The viscosity of the blood in narrow capillary tubes," American Journal of Physiology, 96(3), pp. 562–568.
23. Wells RE, Merrill EW (1961) "SHEAR RATE DEPENDENCE OF VISCOSITY OF WHOLE BLOOD AND PLASMA," Science, 133(345), pp. 763–&.
24. Biferale L (2008) "A note on the fluctuation of dissipative scale in turbulence," Physics of Fluids, 20(3).
25. Pries AR, Secomb TW, Gessner T, Sperandio MB, Gross JF, et al. (1994) "Resistance to blood flow in microvessels in vivo," Circ Res, 75(5), pp. 904–915.
26. Schumacher J (2007) "Sub-Kolmogorov-scale fluctuations in fluid turbulence," Epl, 80(5).
27. Yakhot V (2006) "Probability densities in strong turbulence," Physica D-Nonlinear Phenomena, 215(2), pp. 166–174.
28. Yakhot V, Sreenivasan KR (2005) "Anomalous scaling of structure functions and dynamic constraints on turbulence simulations," Journal of Statistical Physics, 121(5–6), pp. 823–841.
29. Bacher RP, Williams MC (1970) "HEMOLYSIS IN CAPILLARY FLOW," Journal of Laboratory and Clinical Medicine, 76(3), pp. 485–&.
30. Rooney JA (1970) "HEMOLYSIS NEAR AN ULTRASONICALLY PULSATING GAS BUBBLE," Science, 169(3948), pp. 869–&.
31. Fischer TM (1980) "On the energy dissipation in a tank-treading human red blood cell," Biophys. J., 32(2), pp. 863–868.
32. Tran-Son-Tay R, Sutera S, Rao P (1984) "Determination of red blood cell membrane viscosity from rheoscopic observations of tank-treading motion," Biophys,J., 46(1), pp. 65–72.
33. Hellums JD (1994) "1993 Whitaker Lecture: biorheology in thrombosis research," Ann. Biomed. Eng., 22(5), pp. 445–455.
34. Brouwers JJH (2007) "Dissipation equals production in the log layer of wall-induced turbulence," Physics of Fluids, 19(10).
35. Forleo M, Dasi L (2013) "Effect of Hypertension on the Closing Dynamics and Lagrangian Blood Damage Index Measure of the B-Datum Regurgitant Jet in a Bileaflet Mechanical Heart Valve," Annals of Biomedical Engineering, pp. 1–13.
36. Dasi LP, Ge L, Simon HA, Sotiropoulos F, Yoganathan AP (2007) "Vorticity dynamics of a bileaflet mechanical heart valve in an axisymmetric aorta," Physics of Fluids, 19(6).
37. Morshed KN, Dasi LP (2013) "Effect of strong anisotropy on the dissipative and non-dissipative regimes of the second-order structure function," Experiments in Fluids, 54(5).
38. Bluestei M, Mockros LF (1969) "HEMOLYTIC EFFECTS OF ENERGY DISSIPATION IN FLOWING BLOOD," Med. Biol. Eng., 7(1), pp. 1–&.
39. Jones SA (1995) "A RELATIONSHIP BETWEEN REYNOLDS STRESSES AND VISCOUS DISSIPATION - IMPLICATIONS TO RED-CELL DAMAGE," Ann. Biomed. Eng., 23(1), pp. 21–28.
40. Falati S, Gross P, Merrill-Skoloff G, Furie BC, Furie B (2002) "Real-time in vivo imaging of platelets, tissue factor and fibrin during arterial thrombus formation in the mouse," Nat. Med., 8(10), pp. 1175–1180.
41. Ham TH, Shen SC, Fleming EM, Castle W (1948) "Studies on the destruction of red blood cells. iv thermal injury: action of heat in causing increased spheroidicity, osmotic and mechanical fragilities and hemolysis of erythrocytes; observations on the mechanisms of destruction of such erythrocytes in dogs and in a patient with a fatal thermal burn," Blood, 3(4), pp. 373–403.

42. Gershfeld N, Murayama M (1988) "Thermal instability of red blood cell membrane bilayers: temperature dependence of hemolysis," The Journal of membrane biology, 101(1), pp. 67–72.
43. Helms CC, Marvel M, Zhao W, Stahle M, Vest R, et al. "Mechanisms of hemolysis-associated platelet activation," J. Thromb. Haemost., 11(12), pp. 2148–2154.
44. Nesbitt WS, Westein E, Tovar-Lopez FJ, Tolouei E, Mitchell A, et al. (2009) "A shear gradient-dependent platelet aggregation mechanism drives thrombus formation," Nat Med, 15(6), pp. 665–673.
45. Shankaran H, Alexandridis P, Neelamegham S (2003) "Aspects of hydrodynamic shear regulating shear-induced platelet activation and self-association of von Willebrand factor in suspension," Blood, 101(7), pp. 2637–2645.
46. Schneider, S. W., Nuschele, S., Wixforth, A., Gorzelanny, C., Alexander-Katz, A., Netz, R. R., and Schneider, M. F., 2007, "Shear-induced unfolding triggers adhesion of von Willebrand factor fibers," Proc. Natl. Acad. Sci. U. S. A., 104(19), pp. 7899–7903.
47. Siedlecki CA, Lestini BJ, Kottke-Marchant KK, Eppell SJ, Wilson DL, et al. (1996) "Shear-dependent changes in the three-dimensional structure of human von Willebrand factor," Blood, 88(8), pp. 2939–2950.
48. Colace TV, Diamond SL "Direct observation of von Willebrand factor elongation and fiber formation on collagen during acute whole blood exposure to pathological flow," Arterioscler. Thromb. Vasc. Biol., 33(1), pp. 105–113.
49. Harrison P, Cramer EM (1993) "Platelet alpha-granules," Blood Reviews, 7(1), pp. 52–62.
50. Ghasemzadeh M, Kaplan ZS, Alwis I, Schoenwaelder SM, Ashworth KJ, et al. (2013) "The CXCR1/2 ligand NAP-2 promotes directed intravascular leukocyte migration through platelet thrombi," Blood, 121(22), pp. 4555–4566.
51. Nomura S, Tandon NN, Nakamura T, Cone J, Fukuhara S, et al. (2001) "High-shear-stress-induced activation of platelets and microparticles enhances expression of cell adhesion molecules in THP-1 and endothelial cells," Atherosclerosis, 158(2), pp. 277–287.
52. Houk AR, Jilkine A, Mejean CO, Boltyanskiy R, Dufresne ER, et al. (2012) "Membrane Tension Maintains Cell Polarity by Confining Signals to the Leading Edge during Neutrophil Migration," Cell, 148(1–2), pp. 175–188.
53. Gauthier NC, Masters TA, Sheetz MP (2012) "Mechanical feedback between membrane tension and dynamics," Trends Cell Biol.
54. Bark Jr DL, Ku DN (2010) "Wall shear over high degree stenoses pertinent to atherothrombosis," J. Biomech., 43(15), pp. 2970–2977.
55. Geisen U, Heilmann C, Beyersdorf F, Benk C, Berchtold-Herz M, et al. (2008) "Non-surgical bleeding in patients with ventricular assist devices could be explained by acquired von Willebrand disease," Eur. J. Cardiothorac. Surg., 33(4), pp. 679–684.
56. Meyer AL, Malehsa D, Bara C, Budde U, Slaughter MS, et al. (2010) "Acquired von Willebrand syndrome in patients with an axial flow left ventricular assist device," Circulation: Heart Failure, 3(6), pp. 675–681.
57. Gardiner EE, Andrews RK "Platelet Receptor Expression and Shedding: Glycoprotein Ib-IX-V and Glycoprotein VI," Transfus. Med. Rev.
58. Zydney AL, Colton CK (1988) "Augmented solute transport in the shear flow of a concentrated suspension," PhysicoChem. Hydrodyn., 10(1), pp. 77–96.
59. Bark Jr DL, Ku DN (2013) "Platelet Transport Rates and Binding Kinetics at High Shear over a Thrombus," Biophys. J., 105(2), pp. 502–511.
60. Hubbell JA, McIntire LV (1986) "Platelet active concentration profiles near growing thrombi. A mathematical consideration," Biophys. J., 50(5), pp. 937–945.
